# Drug Repurposing of the Anthelmintic Niclosamide to Treat Multidrug-Resistant Leukemia

**DOI:** 10.3389/fphar.2017.00110

**Published:** 2017-03-10

**Authors:** Sami Hamdoun, Philipp Jung, Thomas Efferth

**Affiliations:** Department of Pharmaceutical Biology, Institute of Pharmacy and Biochemistry, Johannes Gutenberg UniversityMainz, Germany

**Keywords:** chemotherapy, pharmacogenomics, drug resistance, transcription factors, oxidative stress

## Abstract

Multidrug resistance, a major problem that leads to failure of anticancer chemotherapy, requires the development of new drugs. Repurposing of established drugs is a promising approach for overcoming this problem. An example of such drugs is niclosamide, a known anthelmintic that is now known to be cytotoxic and cytostatic against cancer cells. In this study, niclosamide showed varying activity against different cancer cell lines. It revealed better activity against hematological cancer cell lines CCRF-CEM, CEM/ADR5000, and RPMI-8226 compared to the solid tumor cell lines MDA-MB-231, A549, and HT-29. The multidrug resistant CEM/ADR5000 cells were similar sensitive as their sensitive counterpart CCRF-CEM (resistance ration: 1.24). Furthermore, niclosamide caused elevations in reactive oxygen species and glutathione (GSH) levels in leukemia cells. GSH synthetase (GS) was predicted as a target of niclosamide. Molecular docking showed that niclosamide probably binds to the ATP-binding site of GS with a binding energy of -9.40 kcal/mol. Using microscale thermophoresis, the binding affinity between niclosamide and recombinant human GS was measured (binding constant: 5.64 μM). COMPARE analyses of the NCI microarray database for 60 cell lines showed that several genes, including those involved in lipid metabolism, correlated with cellular responsiveness to niclosamide. Hierarchical cluster analysis showed five major branches with significant differences between sensitive and resistant cell lines (*p* = 8.66 × 10^5^). Niclosamide significantly decreased nuclear factor of activated T-cells (NFAT) activity as predicted by promoter binding motif analysis. In conclusion, niclosamide was more active against hematological malignancies compared to solid tumors. The drug was particularly active against the multidrug-resistant CEM/ADR5000 leukemia cells. Inhibition of GSH synthesis and NFAT signaling were identified as relevant mechanisms for the anticancer activity of niclosamide. Gene expression profiling predicted the sensitivity or resistance of cancer cells to niclosamide.

## Introduction

Niclosamide, an anthelmintic drug that has been used for about 50 years, is known to be safe and well tolerated. Niclosamide has been identified as a potential anticancer agent that exerts cytotoxic and cytostatic activity against a wide range of cancer types, including leukemia, breast cancer, prostate cancer, hepatocellular carcinoma, and glioblastoma. Additionally it has shown anti-invasive and anti-migratory effects. Several signaling pathways are inhibited by niclosamide in cancer cells including the Wnt/β-catenin, mechanistic target of rapamycin complex 1 (mTORC1), signal transducer and activator of transcription 3 (STAT3), nuclear factor kappa-light-chain-enhancer of activated B cells (NF-κB), and Notch pathways (Li et al., 2014).

Multidrug resistance (MDR) is a major problem in cancer patients that leads to failure of chemotherapy. It affects most cancers and is characterized by cross-resistance to a wide range of commonly used chemotherapeutic drugs (Bartsevich and Juliano, 2000). Most cancers consist of a mixture of heterogeneous malignant cells. Some of them are drug-sensitive and others are drug-resistant. As a result, chemotherapeutic agents mostly kill sensitive cells, but leave out a great proportion of resistant cell populations. Therefore, recurrent tumors are frequently resistant with fatal consequences for the patients (Housman et al., 2014). Of the various mechanisms that contribute to MDR in cancer, the increased efflux of drugs by the ATP-binding cassette (ABC) transporters is the most encountered. The most important of these transporters is P-glycoprotein (Pgp; Bellamy, 1996).

Elevated levels of reactive oxygen species (ROS) occur in almost all types of cancer. They are involved in the promotion of tumor development and progression (Storz, 2005). Cancer cells also express increased levels of antioxidants to detoxify ROS. The process of ROS detoxification is facilitated either through antioxidant enzymes, which scavenge different types of ROS, or by non-enzymatic molecules. Antioxidant enzymes include catalase, superoxide dismutase, and peroxiredoxins. Non-enzymatic antioxidants include glutathione (GSH), flavonoids, vitamins A, C, and E (Liou and Storz, 2010). Treatment of cancer cells with vitamin E and vitamin C (ROS scavengers) increased the expression of Pgp. This suggests that Pgp-mediated MDR can be circumvented under conditions of elevated ROS levels. One of the compounds that elevate ROS levels in cancer cells is niclosamide. ROS generation plays an important role in the anticancer activity of niclosamide in acute myeloid leukemia and lung cancer cells (Wartenberg et al., 2005; Jin et al., 2010; Lee et al., 2014).

The ultimate goal of drug development is to identify molecules with the desired effect in the human body and to establish its quality, safety, and efficacy for treating patients (Kraljevic et al., 2004). Drug development, starting with the initial discovery of a promising target to the final marketed medication, is an expensive, lengthy, and incremental process (Hoelder et al., 2012). An alternative approach is drug repositioning or repurposing, in which new indications are found for existing drugs. The advantages of this approach is that the pharmacokinetics, pharmacodynamics, and toxicity profiles of the investigated drugs are already known. If successful, this leads to a great reduction in time and money expenditures for the evaluation of drugs during preclinical and clinical development (Ashburn and Thor, 2004; Tada et al., 2006).

In order to evaluate the usefulness of niclosamide for MDR, we investigated its activity on the sensitive CCRF-CEM and the MDR (Pgp overexpressing) CEM/ADR5000 leukemia cells. As niclosamide is known to elevate ROS levels in cancer cells, we also tested its effect on ROS generation in both cell lines. Furthermore, we have attempted to identify the molecular mechanisms of action of niclosamide, by microarray-based analyses.

## Materials and Methods

### Cell-Lines

CCRF-CEM and CEM/ADR5000 leukemia, RPMI-8226 multiple myeloma, and HT-29 colorectal cancer cells were grown in RPMI 1640 medium supplemented with 10% fetal bovine serum (FBS), penicillin (100 U/ml)/streptomycin (100 μg/ml) in a 5% CO_2_ atmosphere at 37°C. MDA-MB-231 breast cancer and A549 lung cancer cells were grown in Dulbecco’s Modified Eagle’s Medium supplemented with 10% FBS, penicillin (100 U/ml)/streptomycin (100 μg/ml) in a 5% CO_2_ atmosphere at 37°C. Cells were passaged twice weekly. Resistance of CEM/ADR5000 was maintained by treatment with 5000 ng/ml doxorubicin for 24 h every 2 weeks. All experiments were performed with cells in the logarithmic growth phase.

### Cytotoxicity Assay

Cells obtained from exponential phase cultures were counted and seeded into 96-well plates. The seeding density was 10^4^ cells per well for both cell lines. Cells were then exposed to niclosamide (Sigma-Aldrich, Taufkirchen, Germany) using 0.001, 0.01, 0.1, 1, 10, and 100 μM, in all cell lines. After a 72 h incubation period, 20 μl of resazurin 0.01% w/v was added to each well and the plates were incubated at 37°C for 4 h. Fluorescence was measured using an Infinite M2000 Pro plate reader (Tecan, Crailsheim, Germany). Dose–response curves were generated by plotting the mean cell viability (%) against the concentration of the compound (μM). IC_50_ values were calculated from a calibration curve by linear regression using Microsoft Excel. The resistance ratio for sensitive CCRF-CEM and resistant CEM/ADR5000 cells was calculated by: IC_50 resistant_/IC_50 sensitive_. Experiments were repeated three times.

### ROS Assay

For each sample 2 × 10^6^ cells were seeded in each well of a six-well plate. Each well was treated with 1.5 μM of niclosamide or dimethyl sulfoxide (DMSO). After 24 h incubation, cells were centrifuged and resuspended in RPMI-1640 culture medium and incubated with 10 μM 2′,7′-dichlorodihydrofluorescein-diacetate (H_2_DCFH-DA) for 20 min in the dark. Subsequently, cells were centrifuged, washed with phosphate buffered saline (PBS), resuspended in culture medium and measured in a BD-C6 flow cytometer (Becton-Dickinson, Heidelberg, Germany). Cells were also treated with H_2_O_2_ for 15 min as a positive control. For each sample, 1 × 10^4^ cells were counted. 2′,7′-dichlorofluorescein (DCF) was measured at 488 nm excitation and detected using a 530/30 nm bandpass filter. The experiment was repeated three times.

### Target Prediction

The structure of niclosamide was obtained from ChemSpider^1^ and saved as a mol file. The compound was then submitted to the DRAR-CPI software^2^. The protein targets showing the highest docking scores were obtained.

### Glutathione Assay

The levels of GSH were determined after the treatment of cells with 0.75, 1.5, 3, 6, and 12 μM niclosamide, and incubated for 24 h at 37°C. The cells were then centrifuged and suspended in PBS supplemented with 5% FBS. The cells were stained with 40 μM monochlorobimane and incubated for 20 min. Fluorescence was read using an LSR-Fortessa flow cytometer (Becton-Dickinson, Heidelberg, Germany) using a 405 nm laser. The experiment was repeated three times.

### Molecular Docking

The PDB file for the crystal structure of GSH synthetase (GS) (PDB ID: 2HGS) was downloaded from the protein data bank^3^. To perform molecular docking, the protein structure of GS were first processed with AutodockTools-1.5.6rc316 to overcome problems of incomplete structures due to missing atoms or water and the presence of multimers or interaction partners of the receptor molecule. The output file after preparation was set in PDBQT format, where information about atomic partial charges, torsion degrees of freedom and different atom types were added, e.g., aliphatic and aromatic carbon atoms or polar atoms forming hydrogen bonds. A grid box was then constructed to define docking spaces. The dimensions of the grid box were set around the entire protein molecule in a manner that the ligand could freely move and rotate in the docking space. The grid box consisted of 126 grid points in all three dimensions (X, Y, and Z) separated by a distance of 1 Å between each one. Energies at each grid point were evaluated for each atom type present in the ligand, and the values were used to predict the energy of a particular ligand configuration. Docking parameters were set to 250 runs and 2,500,000 energy evaluations for each cycle. Docking was performed for niclosamide on GS using Autodock4 by means of a Lamarckian algorithm. The corresponding binding energies and the number of conformations in each cluster were attained from the docking log files (dlg). The process was repeated in a triplicate. The mean and standard deviation were calculated.

### Microscale Thermophoresis

The interaction between recombinant human GS (Abcam, Cambridge, UK) and niclosamide was studied using microscale thermophoresis as previously described (Seo and Efferth, 2016). Protein was labeled according to the Monolith^TM^ NT.115 Protein Labeling Kit BLUE-NHS (Amine reactive; NanoTemper Technologies GmbH, Munich, Germany). The labeled human GS was titrated with niclosamide. The final concentrations of niclosamide were 200, 100, 50, 25, and 3.125 μM in analysis buffer (50 mM Tris buffer pH 7.6 containing 150 mM NaCl, 10 mM MgCl_2_, and 0.05% Tween-20). Samples were analyzed using hydrophilic capillaries in NanoTemper Monolith^TM^ NT (NanoTemper Technologies GmbH, Munich, Germany) for blue dye fluorescence.

### COMPARE Analyses

The mRNA microarray data of the NCI tumor cell line panel available at the NCI website^4^ was used (Scherf et al., 2000; Staunton et al., 2001). COMPARE analyses were performed to produce rank-ordered lists of genes expressed in the NCI cell lines as previously described (Paull et al., 1989; Wosikowski et al., 1997). Briefly, every gene of the NCI microarray database was ranked for similarity of its mRNA expression to the log_10_IC_50_ values for niclosamide. To derive COMPARE rankings, a scale index of correlation coefficients (*R*-values) was created.

### Hierarchical Cluster Analysis

Hierarchical cluster analysis was performed, in order to create a cluster model for the different cell lines. This was done by classifying objects into dendrograms. The distances were calculated according to the closeness of between-individual distances. Cluster models have previously been validated for gene expression profiling and for approaching molecular pharmacology of anticancer agents (Efferth et al., 1997; Scherf et al., 2000).

### Promoter Binding Motif Analysis

Binding motifs for transcription factors in the promoter sequences of genes were analyzed by Cistrome analysis software (Liu et al., 2011). Briefly, genes of interest were retrieved in BED format from https://genome.ucsc.edu/cgi-bin/hgTables. SeqPos^5^ was used to screen for enriched transcription factor binding motifs in gene promoter sequences. The screening was performed for motifs deposited in the Transfac, JASPAR, UniPROBE, and PDI databases. Moreover, *de novo* motifs where identified by using MDscan algorithm.

### Nuclear Factor of Activated T-Cells Reporter Assay

HEK293 cell lines were transfected with nuclear factor of activated T-cells (NFAT)-luciferase reporter construct (Qiagen, Germantown, MD, USA). The cells were cultured, according to the manufacturer’s recommendations. The cells were treated with varying concentrations of niclosamide for 24 h. NFAT promoter activity was quantified with Dual-Luciferase Reporter Assay System (Promega, Madison, WI, USA) by measuring the firefly and renilla luciferase luminescences on an Infinite M2000 Pro^TM^ plate reader (Tecan). The ratio of firefly luciferase intensity to renilla luciferase intensity yielded a measure for NFAT activity. The relative luminance for each sample was calculated as: firefly luciferase luminescence/renilla luciferase luminescence. DMSO treatment served as control. Normalized NFAT activity was calculated by the formula: relative luciferase of sample/relative luciferase of the DMSO control. The experiment was repeated three times.

### Statistical Analysis

Data were expressed as mean ± SD of three independent tests. Statistical significance was determined using the Student’s *t*-test. A *p*-value of less than 0.05 denoted significance in all cases. Hierarchical cluster analysis was performed using the Ward’s method (WinSTAT program, Kalmia, Cambridge, MA, USA).

## Results

### Cytotoxicity Assay

**FIGURE 1:**
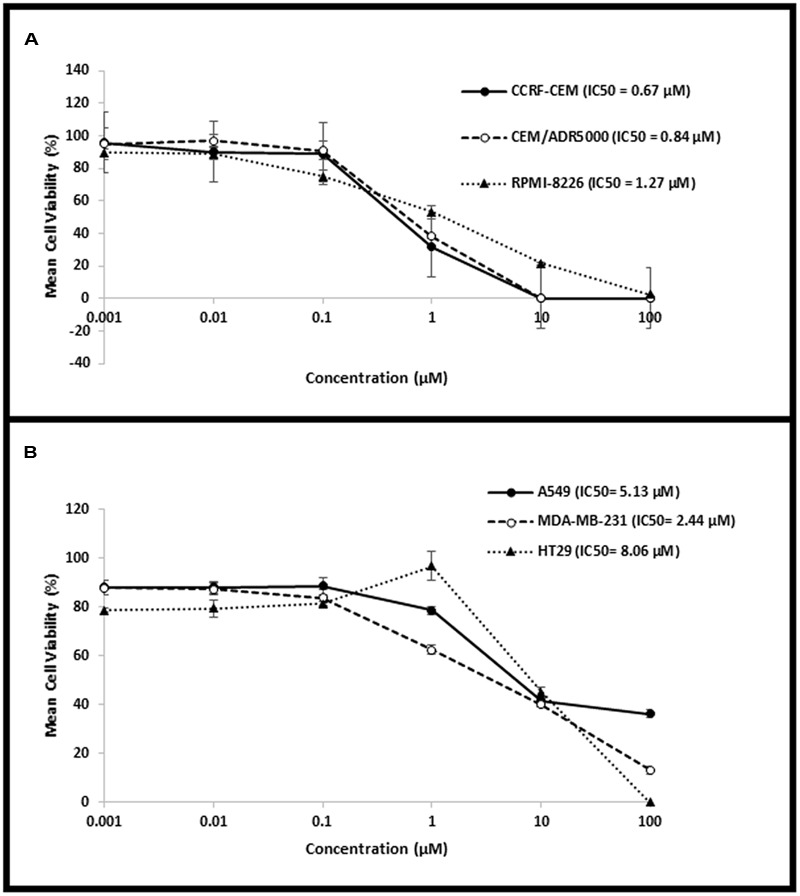
**Dose–response curves from the cytotoxicity assays of (A)** hematological cancer cell lines (CCRF-CEM, CEM/ADR5000, and RPMI-8226) and **(B)** solid tumor cell lines (HT-29, MDA-MB-231, and A549).

The cytotoxic activity of niclosamide was tested on CCRF-CEM and CEM/ADR5000 leukemia, RPMI-8226 multiple myeloma, HT-29 colorectal cancer, MDA-MB-231 breast cancer, and A549 lung cancer cells, using resazurin assay. Niclosamide showed varying activity towards the tested cell lines as shown in **Figure 1**. It was most active against the hematological cancer cell lines CCRF-CEM, CEM/ADR5000, and RPMI-8226, compared to the solid tumor cell lines HT-29, MDA-MB-231, and A549. The resistance ratio between the sensitive CCRF-CEM and multidrug resistant CEM/ADR5000 cells was 1.24, indicating that CEM/ADR5000 were sensitive to niclosamide.

### ROS Assay

**FIGURE 2:**
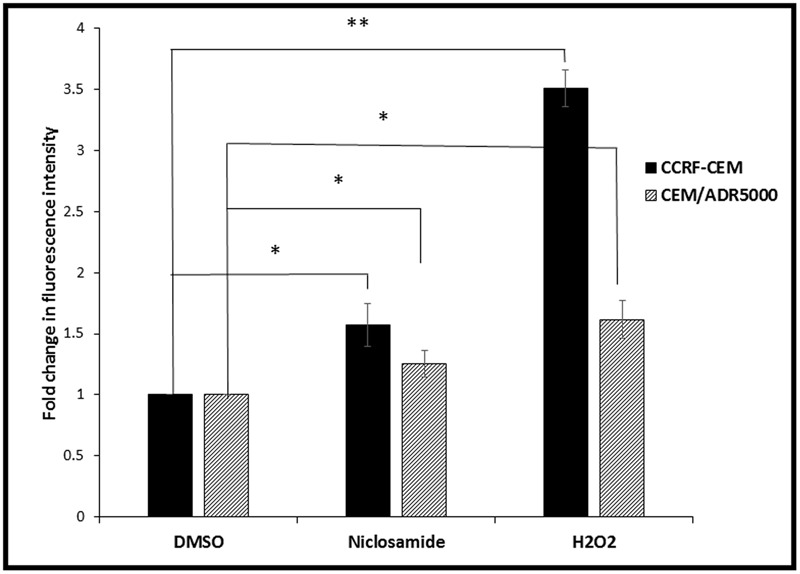
**Effect of 24 h treatment with niclosamide (1.5 μM) on ROS levels in CCRF-CEM and CEM/ADR5000 cells.** H_2_O_2_ (50 μM) was used as a positive control (^∗^*p* < 0.05, ^∗∗^*p* < 0.01, compared to DMSO-treated control cells).

Cellular ROS levels were analyzed after niclosamide treatment by H_2_DCFH-DA staining and flow cytometry. A clear dose-dependent increase in cellular ROS levels was observed after 24 h incubation with niclosamide (**Figure 2**). Thus, niclosamide can be regarded as a ROS inducer in acute lymphoblastic leukemia cells.

### Target Prediction

A total of 391 biological targets were identified for niclosamide by the DRAR-CPI algorithm. Interestingly, GS, which is directly involved in ROS metabolism, was among the predicted targets. Niclosamide showed a docking score of -47.8 and a *Z*′-score of -0.788 for GS. We therefore hypothesized that the binding of niclosamide to GS and the subsequent inhibition of GSH production played an important role in the increase of ROS after treatment of cells.

### Glutathione Assay

**FIGURE 3:**
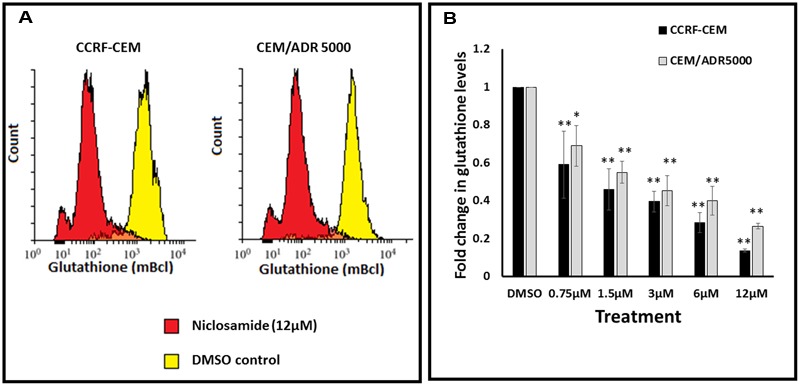
**Effect of niclosamide on glutathione levels in CCRF-CEM and CEM/ADR5000 cells. (A)** Flow cytometric analysis of glutathione levels after treatment with niclosamide (12 μM) for 24 h. **(B)** Statistical quantification of glutathione levels after treatment with different concentrations of niclosamide (^∗^*p* < 0.05, ^∗∗^*p* < 0.01, compared to DMSO-treated control cells).

In order to test whether niclosamide affected GSH levels in the sensitive and resistant cell lines, GSH assays were performed after treatment of leukemia cells with different concentrations of niclosamide. The cells were stained with monochlorobimane and fluorescence was measured using flow cytometry. As shown in **Figure 3**, niclosamide significantly reduced the intracellular GSH levels in a dose-dependent manner in both cell lines. However, the effect on sensitive CCRF-CEM cells was slightly higher than the effect on the resistant CEM/ADR5000 cells, which is consistent with the results from the ROS assay.

### Molecular Docking

**FIGURE 4:**
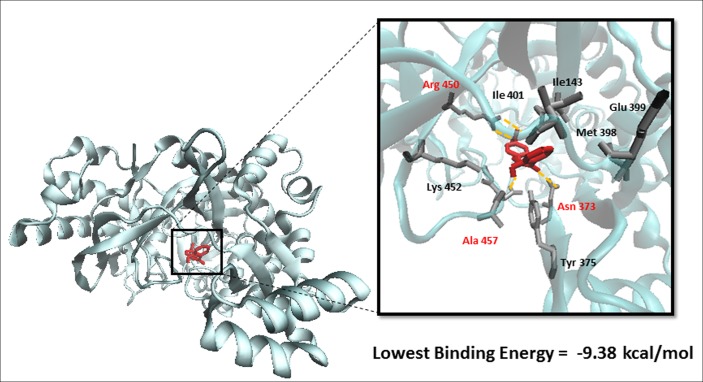
**Binding of niclosamide at the ATP binding domain of glutathione synthetase (GS).** Hydrogen bond forming amino acids are shown in red.

To predict the binding affinity of niclosamide to GS and propose its binding site, we performed molecular docking. The protein structure of GS was downloaded in PDB format, processed using Autodock Tools and finally converted to PDBQT format. A grid box was then constructed. Energies at each grid point were then evaluated for each atom type present in the ligand. The values were used to predict the energy of a particular ligand configuration. Docking was performed for niclosamide on GS with Autodock4 using the Lamarckian Algorithm. The corresponding binding energies and the number of conformations in each cluster were attained from the docking log files (dlg). As shown in **Figure 4**, the lowest binding energy for niclosamide on GS was predicted to be -9.40 ± 0.01 kcal/mol, which is a considerably low value. The amino acids involved in the interaction included Ile143, Asn373, Tyr375, Met398, Glu399, Ile401, Arg450, Lys452, and Ala457. Two of the amino acids (Arg450 and Ala457) showed interactions with hydrogen bonding.

### Microscale Thermophoresis

**FIGURE 5:**
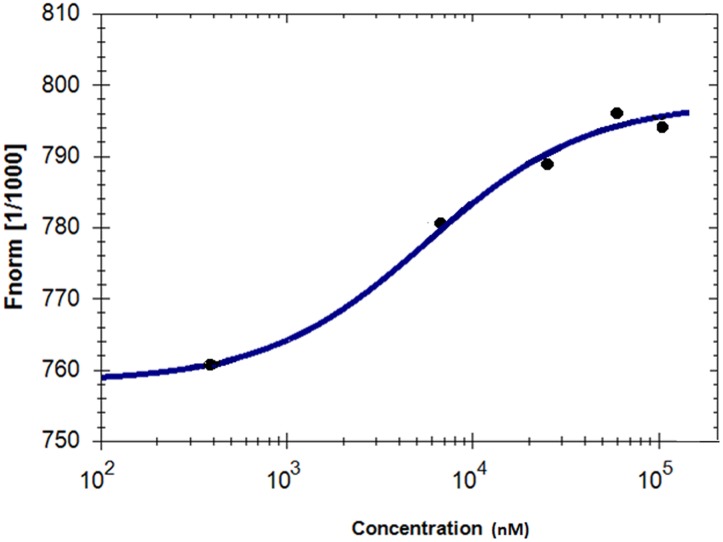
**Characterization of binding affinity of niclosamide labeled GS using microscale thermophoresis**.

Microscale thermophoresis was used to analyze the direct interaction between GS and niclosamide (**Figure 5**). This method is used to determine the binding affinity between a fluorescently labeled protein and a non-labeled compound. Labeled GS was titrated with different concentrations of niclosamide. An equilibrium binding constant of 5.64 μM was obtained providing evidence for direct binding of GS to niclosamide.

### Microarray Analysis

**FIGURE 6:**
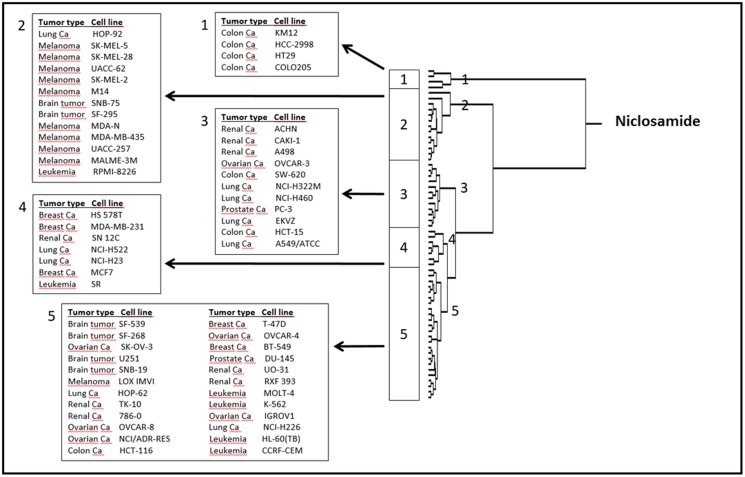
**Dendrogram of hierarchical cluster analysis (Ward’s method) obtained from microarray-based mRNA expression profiles of genes obtained from the NCI database correlating with niclosamide**.

To correlate the cellular responses of niclosamide with the expression of the deregulated genes, we performed COMPARE analyses. Using the NCI database, we correlated the microarray-based transcriptome-wide mRNA expression of 60 tumor cell lines with the log_10_IC_50_ values for niclosamide. We ran a standard COMPARE, which correlated the lowest IC_50_ values with the lowest mRNA expression levels of genes. We then ran a reverse COMPARE which correlated the lowest IC_50_ values with the highest gene expression level. The threshold for correlation coefficients were *R* > 0.55 for standard COMPARE and *R* < -0.55 for reverse COMPARE (Supplementary Table S1). The genes that showed good correlation with sensitivity to niclosamide included those involved in signal transduction (*TP53INP2, LAMTOP5, PDE6G*), lipid metabolism (*SOAT, GMA2*), regulation of cell growth and development (*MAP6, TANC2*) and others. On the other hand, genes that correlated with resistance included those involved in signal transduction (*MUC13, S100P, ARHGEF5, LMO7*), lipid metabolism and transport (*PLA2G2A, CYP3A4, APOM*), protein synthesis (*RPS16, E1F2S2*) and others. The mRNA expression values of these genes were used to perform a hierarchical cluster analysis. The dendrogram of this cluster analysis could be divided into five main cluster branches (**Figure 6**). Sensitivity or resistance to niclosamide and its derivative were predicted by the distribution of cell lines in the dendrogram according to their gene expression profiles. We found a significant difference in the distribution of sensitive and resistant cell lines between the branches of the dendrogram (*p* = 8.66 × 10^-5^). The response of this cell line panel to niclosamide and its derivative can therefore be determined by the gene expression profile.

### Promotor Binding Motif Analysis

To further determine the transcription factors and the signaling pathways involved in the anticancer activity of niclosamide, we performed promotor binding motif analysis. To accomplish that, a set of 30 deregulated genes from the microarray data were selected. As shown in **Table 1**, several transcription factors might be involved in the cellular response of cancer cells to niclosamide. Among them were *CEBPA* and *CEBPB* (cell cycle regulation), *RELA* and *CREL* (NF-κB pathway), *TCF7L1* (Wnt/β-catenin signaling pathway), *SMAD3* [transforming growth factor beta (TGF-β) signaling], *FOXO1* and *NFAT*, all of which are involved in cancer initiation and progression.

**Table 1:** Transcription factors for which promoter motifs are found in the genes identified by COMPARE analysis.

Clusters	Factor	*Z*-score	-10^∗^log (*p*-value)	Clusters	Factor	*Z*-score	-10^∗^log (*p*-value)
1.	EmBP-1b	-4.81	140.979	18.	Pax-4	-3.543	85.274
	Opaque-2	-3.995	103.405	19.	MAF	-3.523	84.535
2.	ZNF766	-4.459	123.985	20.	RFX1	-3.496	83.506
3.	GCR1	-4.445	123.338	21.	THRAP6	-3.443	81.539
4.	CEBPB	-4.158	110.418	22.	bZIP911	-3.374	79.016
5.	REL	-4.09	107.455	23.	Smad3	-3.35	78.123
	c-Rel	-3.438	81.363	24.	NAC69-1	-3.347	78.039
6.	Gat4	-4.083	107.159	25.	FOXO1	-3.337	77.664
7.	TCF7L1	-4.025	104.642	26.	E2A|TCF3	-3.292	76.045
8.	Zfp105	-3.969	102.289	27.	PBF1	-3.288	75.932
9.	Tcfap2e	-3.95	101.515	28.	POU6F1	-3.253	74.699
10.	CEBPA	-3.808	95.674	29.	NF-AT	-3.246	74.44
11.	IRF-2	-3.789	94.879		ESE1|ELF3	-3.177	72.038
12.	ATF6	-3.688	90.89	30.	Elk1	-3.201	72.867
	ROM	-3.482	82.969	31.	PTF1-beta	-3.196	72.707
13.	CPRF-1	-3.367	78.765	32.	Zic2	-3.164	71.599
14.	CPRF-3	-3.204	72.955	33.	GCR1	-3.119	70.039

### NFAT Reporter Assay

**FIGURE 7:**
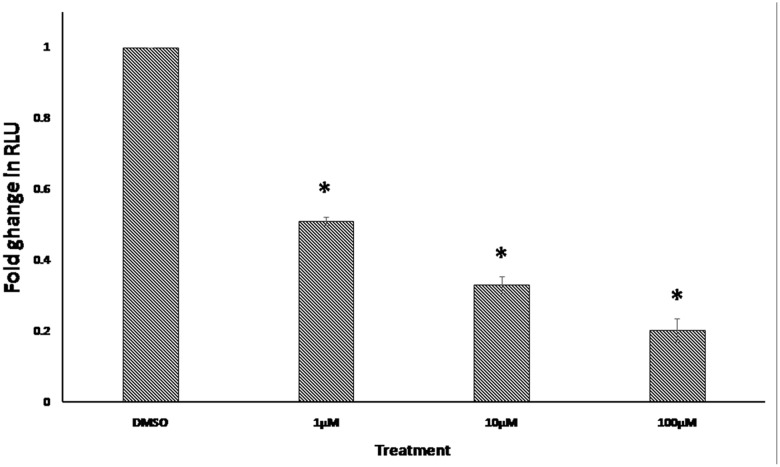
**Effect of 24 h treatment with various concentrations of niclosamide on NFAT signaling activity.** Results shown are the mean values ± SD of three independent experiments (^∗^*p* < 0.01, compared to DMSO-treated control cells).

The NFAT was among the transcription factors that may potentially bind to the gene promoters of deregulated genes from the microarray data. In order to confirm this finding, we performed an NFAT reporter assay. As shown in **Figure 7**, niclosamide indeed caused a significant decrease in NFAT activity in a dose dependent manner.

## Discussion

A major problem of anticancer therapy is the development of MDR. Pgp, an energy dependent efflux pump, plays an important role in the development of MDR. Clinical studies have shown that the overexpression of Pgp in cancer cells is associated with poor prognosis (Bellamy, 1996). This protein transports chemotherapeutic drugs that are central to many anticancer regimens (Allen et al., 2000). Niclosamide showed cytotoxic effects against various types of cancer (Li et al., 2014). Our cytotoxicity assay results on the hematological, breast, lung, and colorectal cancer cell lines, showed that niclosamide is more active against the three hematological cell lines, compared to the solid tumor cell lines cell lines, which were more resistant. However, to the best of our knowledge, the effect of niclosamide on MDR cancer has never been investigated, as of yet. Interestingly we have found niclosamide to be active against both the sensitive and MDR (Pgp-overexpressing) leukemia cells. The resistance ratio was found to be 1.24, indicating that niclosamide shows significant cytotoxic activity against leukemia cells that display the MDR phenotype. The reason may be due to its rapid uptake and the effective bypassing of Pgp, leading to its higher intracellular accumulation and effectiveness. To the best of our knowledge, we report for the first time that niclosamide is active against MDR cancer cells.

ROS are highly reactive chemical entities including radicals, ions or molecules that have a single unpaired electron in their outermost shell of electrons (Liou and Storz, 2010). The use of agents that significantly increase ROS represents an effective strategy to kill cancer cells. Thus, a common approach to treat cancer represents the application of agents with strong pro-oxidant properties. Such agents would either directly generate ROS or inhibit the antioxidant systems in the cell. This will lead to an increase of ROS levels above the threshold, with the subsequent induction of apoptosis and cell death (Ivanova et al., 2013). Niclosamide is known to increase ROS levels in cancer cells, including acute myeloid leukemia and lung cancer cells (Wartenberg et al., 2005; Jin et al., 2010; Lee et al., 2014; Liao et al., 2015). However, the mechanism behind this effect has not been previously investigated. Our results showed that niclosamide also caused the elevation of ROS levels in both the sensitive and MDR leukemia cells. In an effort to elucidate the mechanism by which niclosamide elevated ROS levels, we identified GS as a possible target of niclosamide. GS is a key role player in GSH synthesis, as it catalyzes the formation of GSH from γ-glutamylcysteine and glycine, and therefore determines the overall GSH synthetic capacity in certain tissues, especially under stressful conditions (Lu, 2013). GSH is one of the most important antioxidants in the cell. It plays several vital roles including the maintenance of the redox state, drug detoxification, and cellular protection from damage by free radicals, peroxides, and toxins (Singh et al., 2012). It is involved in DNA repair and apoptosis (Ichijo et al., 1997; Izbicka et al., 1997; Harwaldt et al., 2002), and is clearly associated with cancer resistance to chemotherapeutic agents (Capron et al., 2001; Harwaldt et al., 2002). Niclosamide should therefore reduce the GSH levels in cancer cells. Accordingly, we measured GSH levels in leukemia cells after treatment with different concentrations of niclosamide. The significant reduction in GSH levels indicates an inhibition of GSH synthesis. As GSH is known to be involved in the development of drug resistance (Townsend and Tew, 2003), the inhibition of its synthesis probably plays a role in the activity of niclosamide on MDR cells.

Molecular docking was used to confirm and clarify the inhibitory effect of niclosamide on GS. This bioinformatical tool is considered essential and valuable in drug discovery and development. It gains its value from its ability to predict the conformation of small-molecule ligands within the appropriate target-binding site. It also estimates the ligand receptor binding free energy involved in the molecular interaction (Meng et al., 2011). Niclosamide gave a low binding energy indicating a high affinity to GS. In order to validate the molecular docking result, we studied the real interaction between GS and niclosamide, using microscale thermophoresis. The results showed good binding affinity, confirming our findings. We can therefore postulate that niclosamide is a possible inhibitor of GS. Niclosamide is therefore expected to inhibit the synthesis of GSH in cancer cells causing a reduction of GSH levels shown by the GSH assays. This then leads to the reduction in the ROS scavenging effects of GSH, causing the increased accumulation of ROS in cancer cells. A crystallographic study of the interaction of GS with ATP revealed that Ile143, Tyr375, Met398, Glu399, Ile401, and Lys452 contribute to the interaction between GS and ATP (Polekhina et al., 1999; Dinescu et al., 2004). Accordingly, niclosamide inhibits GS activity by competitively binding to the ATP binding site, and therefore blocks the interaction with ATP. Interestingly GS inhibitors have not been described in previous studies. Niclosamide is therefore the first compound to cause inhibition of GS.

The genes identified by the microarray-based COMPARE analyses that showed good correlation with the cellular response of niclosamide included those involved in lipid metabolism, signal transduction, regulation of cell growth and development, protein synthesis, and others. The result of the hierarchical cluster analysis showed a significant difference in the distribution of sensitive and resistant cell lines between the branches of the dendrogram. The response of this cell line panel to niclosamide can therefore be determined by the gene expression profile. Inhibitors of lipogenic enzymes are quite active and efficient anticancer agents. Several other compounds that target lipid and cholesterol metabolism and homeostasis have shown relevant anticancer activity (Beloribi-Djefaflia et al., 2016). According to our findings from the microarray data analysis, lipid metabolism might play a central role in the cytotoxic activity of niclosamide against cancer cells. Further studies are required to confirm this mechanism of action. These would include the determination of the effect of niclosamide on the levels of lipogenic enzymes and on the amounts of total lipid, total cholesterol and triglyceride in cancer cells. It is worth noting that none of the ABC transporters was found to be associated with resistance to niclosamide. This is consistent with our finding that overexpression of Pgp is not involved in the resistance to niclosamide. The promoter binding motif analysis of the microarray data of niclosamide showed that a number of transcription factors might potentially bind to these gene promoters. Those that are related to cancer include *CEBPA, CEBPB, RELA, CREL, TCF7L1, NFAT*, and *SMAD3*. Thus, several signaling pathways would be involved in the anticancer activity of niclosamide. These pathways include the NF-κB, Wnt, NFAT, TGF-β, and forkhead box protein O (FOXO) signaling pathways. In addition, the CCAAT/enhancer-binding protein (CEBP) transcription factors, involved in cell cycle regulation, might also play a role in the activity of niclosamide. In accordance with our findings, niclosamide is known to exert anticancer activity through inhibition of NF-κB and Wnt signaling pathways (Li et al., 2014). Niclosamide is also known to inhibit mechanistic target of rapamycin (mTOR) signaling (Fonseca et al., 2012). The mTOR signaling pathway regulates FOXO activity as well as TGF-β signaling (Mori et al., 2014; Yu et al., 2015). Furthermore, it controls the ratio of CEBP isoform expression. It is therefore possible that the effect of niclosamide on the expression of FOXO, TGF-β, and CEBP regulated genes is through the inhibition of mTOR signaling.

Previously, the effect of niclosamide on several signaling pathways has been investigated (Li et al., 2014). Even though, its effect on NFAT activity has not been studied yet. The NFAT signaling pathway has an important role in the development and function of the immune system. It is also involved in the development of cardiac, skeletal muscle, and nervous systems. This pathway is activated by increased calcium levels, resulting from its release from the endoplasmic reticulum or its influx through activated channels in the cell membrane. Recent findings have shown that NFAT contributes to cancer development and progression, including solid tumors and hematological malignancies. NFAT signaling is also known to be persistently active in mouse models of human leukemia (Medyouf and Ghysdael, 2008). Inhibition of NFAT signaling pathway in leukemia cells caused cell growth arrest and induction of apoptosis *in vitro* and *in vivo* (Mancini and Toker, 2009). Due to the importance of NFAT signaling in the progression of leukemia and our finding from the promoter binding motif analysis, we further investigated the effect of niclosamide on NFAT signaling using a reporter cell line. We found for the first time that niclosamide significantly inhibited NFAT signaling in a dose-dependent manner. The inhibition of NFAT activity may therefore lead to growth arrest and induction of apoptosis, participating in the anticancer activity of niclosamide.

We conclude that niclosamide exhibits a great potential as an anticancer agent. In this study niclosamide showed excellent activity against MDR leukemia. Niclosamide may therefore have the potential to solve the problem of MDR in cancer patients. The findings of the present study indicate that the cytotoxic activity of niclosamide is due to its targeting of several signaling pathways in cancer cells. We identified the inhibition of GSH synthesis and NFAT signaling as novel mechanisms for the anticancer activity of niclosamide. The microarray data analyses showed that the cellular response of a cancer type can be predicted by its gene expression profile. These data also suggest the involvement of lipid metabolism in the anticancer activity of niclosamide. It is therefore reasonable to consider niclosamide as a clinical candidate for the treatment of refractory MDR cancers.

## Author Contributions

SH performed the cytotoxicity, reactive oxygen species, glutathione and NFAT reporter assays, promoter binding motif analysis, and wrote the paper. PJ performed the microarray analysis and molecular docking. TE supervised the project and wrote the paper.

## Conflict of Interest Statement

The authors declare that the research was conducted in the absence of any commercial or financial relationships that could be construed as a potential conflict of interest.

## Footnotes

1 http://www.chemspider.com

2 https://cpi.bio-x.cn/drar

3 http://www.rcsb.org/pdb

4 https://dtp.cancer.gov

5 http://cistrome.org

## Abbreviations

ABC: ATP-binding cassette
GS: glutathione synthetase
GSH: glutathione
MDR: multidrug resistance
NFAT: nuclear factor of activated T-cells
Pgp: P-glycoprotein
ROS: reactive oxygen species
