# Supplementary material for: Drug Repurposing of the Anthelmintic Niclosamide to Treat Multidrug-Resistant Leukemia
Source: Front Pharmacol. 2017 Mar 10;8:110. doi: 10.3389/fphar.2017.00110 (PMC5344920; doi:10.3389/fphar.2017.00110)
Supplement: Supplementary file 1 [file Table_1.docx]

Supplementary Material

**Drug Repurposing of the Anthelmintic Niclosamide to Treat Multidrug-Resistant Leukemia**

**Sami Hamdoun, Philipp Jung, Thomas Efferth^*^**

Department of Pharmaceutical Biology, Institute of Pharmacy and Biochemistry, Johannes Gutenberg University, Mainz, Germany

* Corresponding Author: Professor Dr. Thomas Efferth, Department of Pharmaceutical Biology, Institute of Pharmacy and Biochemistry, Johannes Gutenberg University, Staudinger Weg 5, 55128 Mainz, Germany. Tel: +49-6131-3925751; Fax: +49-6131-3923752; Email: efferth@uni-mainz.de

**Suppl. Table 1:** Correlation of gene expression values identified by COMPARE analyses with log_10_IC_50_ values of niclosamide from the NCI database

| **Genes correlated with sensitivity** | | | | | | |
| --- | --- | --- | --- | --- | --- | --- |
| **No.** | **Coefficient** | **Experimental ID** | **GenBank Accession** | **Gene Symbol** | **Name** | **Function** |
| 1 | 0.668 | GC12212 | R20806 | SOAT1 | Sterol O-acyltransferase | Catalyzes the formation of fatty acid-cholesterol esters |
| 2 | 0.569 | GC13924 | N22009 | QKI | Transcribed locus RNA | RNA-binding protein that plays a central role in myelinization |
| 3 | 0.531 | GC18139 | AA007414 | COMMD1 | Copper Metabolism (Murr1) Domain Containing 1 | Regulator of ubiquitination of specific cellular proteins |
| 4 | 0.522 | GC15809 | W49759 | TP53INP2 | Tumor protein p53 inducible nuclear protein | Dual regulator of transcription and autophagy |
| 5 | 0.513 | GC16033 | W74752 | GM2A | GM2 ganglioside activator | Catalyzes the degradation of glycosphingolipids with terminal alpha-galactosyl residues |
| 6 | 0.513 | GC14717 | N62239 | CSMD1 | CUB and Sushi multiple domains | Suppressor of squamous cell carcinomas |
| 7 | 0.496 | GC19355 | AA045202 | MAP6 | Microtubule-associated protein 6 | Microtubule stabilization |
| 8 | 0.484 | GC9733 | AA045759 | SRPX | Sushi-repeat-containing protein, X-linked | Ligand for the urokinase plasminogen activator surface receptor |
| 9 | 0.484 | GC14448 | N38845 | TANC2 | Tetratricopeptide repeat, ankyrin repeat and coiled-coil containing 2 | Regulation of dendritic spines, spatial memory, and embryonic development |
| 10 | 0.478 | GC18930 | AA040361 | LAMTOR5 | Late Endosomal/Lysosomal Adaptor, MAPK And MTOR Activator 5 | Regulator complex, involved in amino acid sensing and activation of mTORC1 |
| 11 | 0.473 | GC14599 | N64806 | PXK | PX domain containing serine/threonine kinase | Binds to and modulates brain Na, K-ATPase subunits |
| 12 | 0.472 | GC17479 | AA059121 | PDE6G | Phosphodiesterase 6G | Regulates the c-Src protein kinase and G-protein-coupled receptor kinase 2 |
| 13 | 0.472 | GC14429 | N48885 | MAG | Myelin associated glycoprotein | Adhesion molecule in postnatal neural development |
| 14 | 0.468 | GC13001 | H41276 | PDZD2 | PDZ domain containing 2 | May be involved in the early stages of prostate tumorigenesis |
| 15 | 0.467 | GC9862 | AA047247 | ATP6V1F | V-ATPase Subunit F | Mediates acidification of eukaryotic intracellular organelles |
| **Genes correlated with resistance** | | | | | | |
|  | **Coefficent** | **Experimental ID** | **GenBank Accession** | **Gene Symbol** | **Name** | **Function** |
| 1 | -0.651 | GC14981 | N63943 | LYZ | Lysozyme | Antibacterial activity against a number of bacterial species |
| 2 | -0.627 | GC10390 | AA053681 | RPS16 | Ribosomal protein S16 | Ribosomal protein; component of the 40S subunit |
| 3 | -0.606 | GC18390 | AA029021 | PLA2G2A | Phospholipase A2, group IIA (platelets, synovial fluid) | Regulation of the phospholipid metabolism in biomembranes |
| 4 | -0.572 | GC10184 | AA045699 | TSPAN8 | Tetraspanin 8 | Cell development, activation, growth and motility. |
| 5 | -0.571 | GC10376 | AA053660 | MUC13 | Mucin 13, cell surface associated | Epithelial and hemopoietic transmembrane mucin; may play a role in cell signaling |
| 6 | -0.565 | GC10288 | AA053016 | S100P | S100 calcium binding protein P | May function as calcium sensor |
| 7 | -0.563 | GC12390 | R70701 | EPSTI1 | Epithelial Stromal Interaction Protein 1 | Up-regulated in breast carcinomas |
| 8 | -0.559 | GC18116 | AA005112 | LMO7 | LIM domain 7 | May be involved in protein-protein interactions |
| 9 | -0.558 | GC17636 | W85901 | EIF2S2 | Eukaryotic translation initiation factor 2, subunit 2 beta | Functions in the early steps of protein synthesis |
| 10 | -0.556 | GC9879 | AA045822 | ARHGEF5 | Rho guanine nucleotide exchange factor 5 | Forms a complex with G proteins; stimulates Rho-dependent signals. |
| 11 | -0.552 | GC10328 | AA053557 | NDUFA9 | NADH dehydrogenase (ubiquinone) 1 alpha subcomplex, 9 | NADH ubiquinone oxidoreductase |
| 12 | -0.547 | GC13060 | R92425 | CYP3A4 | Cytochrome P450 3A3 | Drug metabolism; synthesis of cholesterol, steroids and other lipids |
| 13 | -0.544 | GC12620 | R48163 | ITGB4 | Integrin, beta 4 | Receptor for laminin. |
| 14 | -0.542 | GC10712 | R42245 | FAM169A | Family with sequence similarity 169, member A | Function not identified yet |
| 15 | -0.526 | GC17235 | AA047660 | APOM | Apolipoprotein M | Probably involved in lipid transport. |
